# The prevalence and management of heart failure in Dutch nursing homes; design of a multi-centre cross-sectional study

**DOI:** 10.1186/1471-2318-12-29

**Published:** 2012-06-11

**Authors:** Mariëlle AMJ Daamen, Jan PH Hamers, Anton PM Gorgels, Hans-Peter Brunner-la Rocca, Frans ES Tan, Marja P van Dieijen-Visser, Jos MGA Schols

**Affiliations:** 1 Department of Health Services Research, School for Public Health and Primary Care, Maastricht University, Maastricht, The Netherlands; 2 Department of Cardiology, Maastricht University Medical Centre, Maastricht, The Netherlands; 3 Department of Methodology and Statistics, Maastricht University, Maastricht University, Maastricht, The Netherlands; 4 Department of Clinical Chemistry, Maastricht University Medical Centre, Maastricht, The Netherlands; 5 Department of General Practice, School for Public Health and Primary Care, Maastricht University, Maastricht, The Netherlands

## Abstract

**Background:**

Heart failure is likely to be particularly prevalent in the nursing home population, but reliable data about the prevalence of heart failure in nursing homes are lacking. Therefore the aims of this study are to investigate (a) the prevalence and management of heart failure in nursing home residents and (b) the relation between heart failure and care dependency as well as heart failure and quality of life in nursing home residents.

**Methods/design:**

Nursing home residents in the southern part of the Netherlands, aged over 65 years and receiving long-term somatic or psychogeriatric care will be included in the study. A panel of two cardiologists and a geriatrician will diagnose heart failure based on data collected from actual clinical examinations (including history, physical examination, ECG, cardiac markers and echocardiography), patient records and questionnaires. Care dependency will be measured using the Care Dependency Scale. To measure the quality of life of the participating residents, the Qualidem will be used for psychogeriatric residents and the SF-12 and VAS for somatic residents.

**Conclusion:**

The study will provide an insight into the actual prevalence and management of heart failure in nursing home residents as well as their quality of life and care dependency.

**Trial registration:**

Dutch trial register NTR2663

## Background

Heart failure (HF) is a highly prevalent chronic disease in older persons. Its prevalence increases with age reaching 15–20% in those aged over 80 years [1]. According to current guidelines [2], HF is stated as a clinical syndrome characterized by the presence of symptoms and typical signs of HF and objective evidence of a structural and/or functional abnormality of the heart, usually illustrated by echocardiography [2]. Accurate diagnosis of the presence and aetiology of HF is important given its crucial influence on therapy. However, signs and symptoms of HF in older persons are often obscured, due to physical limitations or the unreliability of clinical history due to dementia [3]. In addition, signs of HF, such as fatigue or dyspnoea, are often attributed to the normal ageing process. Besides, symptoms are often non-specific and can be attributed to other common diseases in older persons such as venous insufficiency or obesity [4]. The diagnosis of HF is especially challenging if co-morbidities are present that share common symptoms of HF are present such as COPD and venous insufficiency [5].

This implicates that the diagnosis of HF is particularly difficult in older persons and nursing home residents. The latter represent a specific group, involving very frail and disabled elderly persons with chronic somatic diseases or progressive dementia, both often being complicated by co-morbidities [6]. The prevalence of HF in nursing home residents is estimated to be 20–25% [3,7]. Furthermore, HF in this specific group is likely to be underdiagnosed, due to the lack of knowledge regarding adequate diagnosis and treatment of HF in this population. This counts the more because nursing home residents are often excluded from clinical and epidemiological studies.

It is well known that HF is accompanied by a high patient and economic burden [8]. HF patients are often re-admitted to hospital, mainly due to periodic episodes of clinical deterioration [9]. In the Netherlands, as well as other Western countries cardiovascular diseases (including HF) account for the highest hospitalisation rate, resulting in high financial costs [10,11].

Notwithstanding the fact that survival of HF has improved in recent decades, once hospitalised for heart failure, 33% of elderly patients die within the following year [12]. Women and older persons experience less improvement in survival, partly because they often suffer from HF with a preserved ejection fraction, but also because they are less likely to receive treatment with B-blockers and ACE-inhibitors [12,13].

HF also leads to an impaired quality of life [14,15]. The symptoms of HF such as fatigue and dyspnoea result in increased care dependency which is accompanied by a decline in health status and quality of life [16]. Therefore, early diagnosis and treatment of HF may prevent the progression of heart failure and lead to improvement in symptoms and quality of life [17]. Furthermore, it is known that older persons are less likely to receive evidence-based treatments for HF [18]. Although ACE- inhibitors have been demonstrated to benefit HF patients, they remain underused for HF treatment in nursing home settings [19].

The aim of this study is to gain an insight into the prevalence and management of HF in nursing home residents and to explore the association between HF, care dependency and quality of life.

The following research questions will be addressed:

1.What is the prevalence of HF in Dutch nursing home residents?

2. How is HF currently treated in nursing home residents both pharmacologically and non-pharmacologically?

3. What is the association between HF and the care dependency and quality of life of nursing home residents?

This article describes the study protocol.

## Methods/design

This multicentre study will follow a cross-sectional design.

### Setting

Five healthcare organisations that provide institutional long-term care in the Southern part of the Netherlands will participate in the study. These healthcare organisations have various nursing home facilities for both chronic somatic and psychogeriatric care. Dutch nursing home care is provided by teams of registered nurses, nurse assistants, paramedical professionals and nursing home physicians, who are employed by the nursing homes themselves. The care can be characterised as continuing, long lasting, systematic and multidisciplinary care (CLSM) that is provided via an integrated care plan after a thorough and integral assessment of the resident’s problems. Nursing homes also have a geriatric rehabilitation function for a substantial number of somatic residents. The nursing home physician is responsible for all medical care and initiates the different therapeutic interventions [20].

### Study population and sample

The study population will consist of nursing home residents who receive long term care on somatic or psychogeriatric wards. Both somatic and psychogeriatric residents will be eligible when over 65 years of age. Residents receiving palliative care or admitted for short-term rehabilitation ( staying < 2 months) will be excluded. The overall number of nursing home residents at the five participating healthcare organisations is about 4500. After excluding the places allocated for rehabilitation and terminal care about 3500 residents will be available for recruitment.

A total of 1000 nursing home residents will be included in the study, with 200 from each participating organisation. Based on the current segmentation of somatic residents (35%) versus psychogeriatric (65%) residents in Dutch nursing homes, we aim to include 70 somatic and 130 psychogeriatric residents from each organisation.

In a feasibility study on somatic wards, we found that about 50% of the approached residents were willing to participate. Reasons given for not participating included: they considered themselves too old, or believed that the investigations would be too burdensome. We also encountered difficulties, when asking legal representatives to make decisions regarding participation on behalf of residents. These preliminary findings were in line with a study conducted by Barnes et al, who also focused on the recruitment of older people in a HF study, in which only 30% of the initially identified participants agreed to participate [21].

Therefore, it will probably be necessary to approach three times the number of residents per healthcare organisation in order to recruit 200 residents per site.

#### Sample size calculation

The calculation of sample size was based on an estimated prevalence of HF in nursing home residents of *p* = 0.20 with a standard deviation of 0.025 and a confidence level (%) of 95%. No power considerations are required, because there is no null hypothesis to be tested. Accordingly, 983 residents should be included in the study; therefore a sample of 1000 residents will be selected.

### Selection of participants

For practical and logistic reasons, nursing home residents living in the facilities with the highest number of residents, will be the first to be recruited per care home organisation. The residents and/or their legal representatives will receive a letter describing the purpose and content of the study. Informed consent will be obtained from the residents themselves or from their legal representatives in the case of psychogeriatric residents or residents with aphasia.

### Ethical considerations

The study protocol complies with the Declaration of Helsinki and has been granted approval from the Medical Ethics Committee of Maastricht University/Academic Hospital Maastricht. (NL33281.068.10/MEC10-3-074). The study is registered in the Dutch trial register (NTR2663).

### Study protocol

#### Training of nursing home physicians

Nursing home physicians (NHP) at each healthcare organisations (four per site) will participate in the study. The physicians will receive a refresher course, from a cardiologist specialising in HF at the Maastricht University Medical Centre, regarding diagnosing HF and performing a structured physical examination. Training will consist of an introduction about signs and symptoms of HF followed by bed-side teaching (3 hours) and a session and lecture about ECG findings in relation to HF (2 hours). The NHP will subsequently visit the University Medical Centre’s out patient HF clinic to refresh their knowledge and practise diagnostic skills related to HF (3 hours).

#### Assessment of heart failure

After training the NHP will start assessing the participants. During the assessment, the study NHP will perform the anamnesis and the physical examination. To ensure that no prior probability of HF is taken into account during the history taking and physical examination, the NHP will not have access to pre-existing information about the residents, with the exception of their name and date of birth. Two research nurses and a NHP/researcher, who will be responsible for gathering data from the medical records and the questionnaires, will record the ECG and take blood samples for the measurement of the NT-pro BNP marker. The ECG will be interpreted by the same study NHP who executed the examination. A qualified (fellow) cardiologist will record the echocardiogram on site, using a mobile echo device with recording capabilities. The entire assessment will be carried out on site in the participating nursing homes.

#### Diagnosis of heart failure

The final diagnosis of HF will be based on all the data collected and will be made by an expert team of two cardiologists and a geriatrician using the definition of HF from the guidelines of the European Society of Cardiology 2008 [2].

To diagnose HF, data will be collected from actual clinical examinations (including history, physical examination, ECG, cardiac markers and echocardiogram) and from the patients’ records (past medical history and medication).

At the end of the assessment all residents will be categorized in one of the following groups:

1.No HF

2. HF, no previous history

3. HF, already known (either overt HF with signs and symptoms or re-compensated HF)

## Measurements

The data required to answer the research questions are listed in Table 1.

**Table 1:** Overview of data collection

**Variable**		**Measurement**	**Specified**
Clinical characteristics			
	Age	Years	
	Sex	Male, female	
	Diagnosis at admission	Data from medical record	ICD-10
	Co-morbidity	Data from medical record	ICD-10
	Medication	Medication description	Non-cardiac medication
	Cognitive impairment		MMSE
Heart failure		Diagnosis of HF	
		- History	*Suffers from:*
			Dyspnoea on exertion, rest dyspnoea, paroxysmal nocturnal dyspnoea
			Palpitations/irregular heartbeat, leg oedema, orthopnoea, fatigue, increased weight
			*Is known to have:*
			Hypertension, myocardial infarction, cardiac arrhythmia, angina pectoris (chest pain)/coronary artery disease, valvular heart disease and/or surgery, CABG, pacemaker, pre-existing heart failure, history of cardiac surgery
		- Physical examination	*Common signs:*
			Anaemic, cyanotic, dyspnoeic, obesity, pulse rate, blood pressure
			*Signs of right heart failure/fluid retention*
			Neck vein distension, increased jugular venous pressure, right ventricular pulsations,
			pulsations of hepar, hepatojugular reflux, hepatomegaly, ascites, leg oedema.
			*Signs of left heart failure/fluid retention*
			Apex palpable, displacement of the apex, pulmonary rales, signs of pleural effusion,
			3rd heart sound, murmurs, tachycardia, irregular heartbeat/ atrial fibrillation
		- ECG	Normal ECG, sinus tachycardia, sinusbradycardia, myocardial infarction, atrial fibrillation, ventricular
			arrhythmia, pathological Q’s, AV-block, micro voltage, QRS > 120 ms or LBTB morphology,
			left ventricular hypertrophy, left bundle branch block, right ventricular hypertrophy
		- Cardiac marker	NT-pro BNP
		- Echocardiography	LV ejection fraction, LV function, end-diastolic diameter, end-systolic diameter, fractional shortening,
			left atrial size, left ventricular wall thickness, valvular structure and function, right ventricular
			hypertrophy, right ventricular dysfunction
		- Data medical record	Blood test, X-thorax
Treatment of heart failure		Medication description	Cardiac medication
		Non pharmacological	Sodium restriction, fluid restriction, supplementary nutrition, physical activity, stop smoking
Cardiovascular risk profile		Cardiovascular risk factors	Smoking, BMI, diabetes mellitus, hypertension, hypercholesterolaemia
Pre-existing heart failure		Data medical record	Specialist that diagnosed heart failure, criteria/ examinations used for diagnoses
Quality of life		Qualidem for psychogeriatric residents	
		SF-12 for somatic residents	
		VAS for somatic residents	
Care dependency		Care dependency scale	

### Demographic data and clinical characteristics

Data regarding general and specific resident characteristics will be gathered for all participants. The variables age, gender, medication, diagnosis on admission and co-morbidity will be retrieved from the medical records. The cognitive status of each resident will be measured using the Mini Mental State Examination (MMSE). This is a valid test of cognitive function including the areas of orientation, registration, attention and calculation, recall, language repetition and complex commands [22].

### Heart failure

#### Physical examination and clinical history

The actual history and physical examination will be recorded on a client record file developed for this study. In somatic residents the history-related questions will be answered by the residents themselves; in psychogeriatric residents these questions will be answered by the nurses responsible for their daily care and/or the family caregiver of the resident.

#### Electrocardiogram (ECG)

The clinical assessment will also include a standard 12-lead ECG, recorded by the research nurse. Abnormal ECG findings will be identified according to predefined criteria.

#### Measurement of N-terminal pro brain natriuretic peptide (NT-pro BNP)

A venous blood sample will be taken to determine the NT-pro BNP concentration. These samples will be analysed in the clinical chemistry laboratory of the Maastricht University Medical Centre on an Elecsys 2010 (Roche Diagnostics, Mannheim, Germany), using the NT-pro BNP assay. A normal concentration of NT- pro BNP <125 pg/ml in an untreated patient has a high negative predictive value for HF [23].

#### Echocardiogram

The echocardiogram will be recorded using a Philips CX50 CompactXtreme Ultrasound System. The echocardiogram will provide essential information about the aetiology and severity of HF.

#### Complementary information from medical records

Additional information will be obtained from the medical records such as main diagnosis on admission, co-morbidity, results of recent blood tests or X-thorax (if available) and currently prescribed medication.

If HF has already been diagnosed, we will search for information in the medical record about who diagnosed HF and on which criteria the diagnosis was based.

### Current treatment of heart failure

To study the current pharmacological treatment of HF in nursing homes data will be collected on cardiac medication prescriptions and data on non-pharmacological treatment will be extracted from the medical and nursing records.

### Care dependency and quality of life

Care dependency will be assessed using the Care Dependency Scale (CDS). The CDS consists of 15 items measuring human needs on a five-point Likert-type scale and the score ranges from 15 (completely dependent on care) to 75 (almost independent of care) [24,25].

Quality of life will be measured using the Short-Form Health Survey 12 item (SF-12) and the Visual Analogue Scale (VAS) for somatic residents. The SF-12 is a short form derived from the SF 36. It has proven validity and it is appropriate for use in studies with a large sample size and studies focusing on patient-based assessments [26]. The visual analogue scale (VAS) subjectively measures the quality of life experienced on a scale from 0–10.

The Qualidem will be used to measure the quality of life in psychogeriatric residents. This scale contains 37 items divided into 9 subscales. It has sufficient validity to be used for research in residential care settings [27,28].

### Statistical analysis

The data will be analysed using SPSS software PASW Statistics 17 and will include descriptive frequency distributions for all variables. Differences between groups will be tested using Student’s T-test or analysis of variance (ANOVA) for continuous (dependent) variables and the Chi-square test (cross-table analysis) and/or multivariable logistic regression analyses for discrete (dependent) variables.

## Discussion

The study will provide insight into the actual prevalence and management of HF in nursing home residents as well as their quality of life and care dependency. In order to improve the care for HF in nursing home residents, first the accuracy to identify HF in nursing home residents has to be assessed. HF is likely to be highly prevalent in this specific given their advanced age and prevalence of cardiovascular diseases. Furthermore, HF is probably underdiagnosed in this group as symptoms are often not recognised or are misinterpreted. It is also known that HF is not always treated according to current guidelines (ESC-guidelines 2008, CBO 2010) [12,19]. Nursing home residents are often excluded from epidemiological and clinical studies and therefore the benefits and adverse effects of medical treatment of HF in nursing home residents have not been properly examined.

Eventually, the results of the study will lead to recommendations for improving the diagnostic process and management of HF in these patients. Moreover the results of the study may contribute to a better quality of care for HF residents.

This study on the prevalence of HF differs from other studies by the type of approach used to diagnose HF. Of those studies, reviewed by Daamen and colleagues [3], most showed a prevalence of about 15–20%, but were based on retrospective searches of medical records. Only one study revealed a prevalence of HF of 45% [29] and in that study HF was diagnosed after a focused clinical assessment by a geriatrician. This will be the approach used in our study. On the other hand this may also be a limitation, as refusal by eligible participants because they consider the examinations too burdensome may result in a low number of participants.

## Abbreviations

CDS, Care Dependency Scale; COPD, Chronic obstructive pulmonary disease; ECG, Electrocardiography; HF, Heart failure; ICD-10, International Classification of Diseases version 10; MMSE, Mini Mental State Examination; NHP, Nursing home physician; NT-pro BNP, N-terminal pro brain natriuretic peptide; SF-12, Study 12-item Short-Form Health Survey; VAS, Visual Analogue Scale.

## Competing interests

None of the authors have any competing interests arising from this research.

## Authors’ contributions

MvdV: nursing home physician – researcher, designed the study and wrote this manuscript. JS and JH: supervised the study design and provided comments on earlier drafts of this paper. AG, HPB, FT and MvD, helped write and revise the manuscript. All authors read and approved the final version of the manuscript.

## Pre-publication history

The pre-publication history for this paper can be accessed here:

http://www.biomedcentral.com/1471-2318/12/29/prepub
